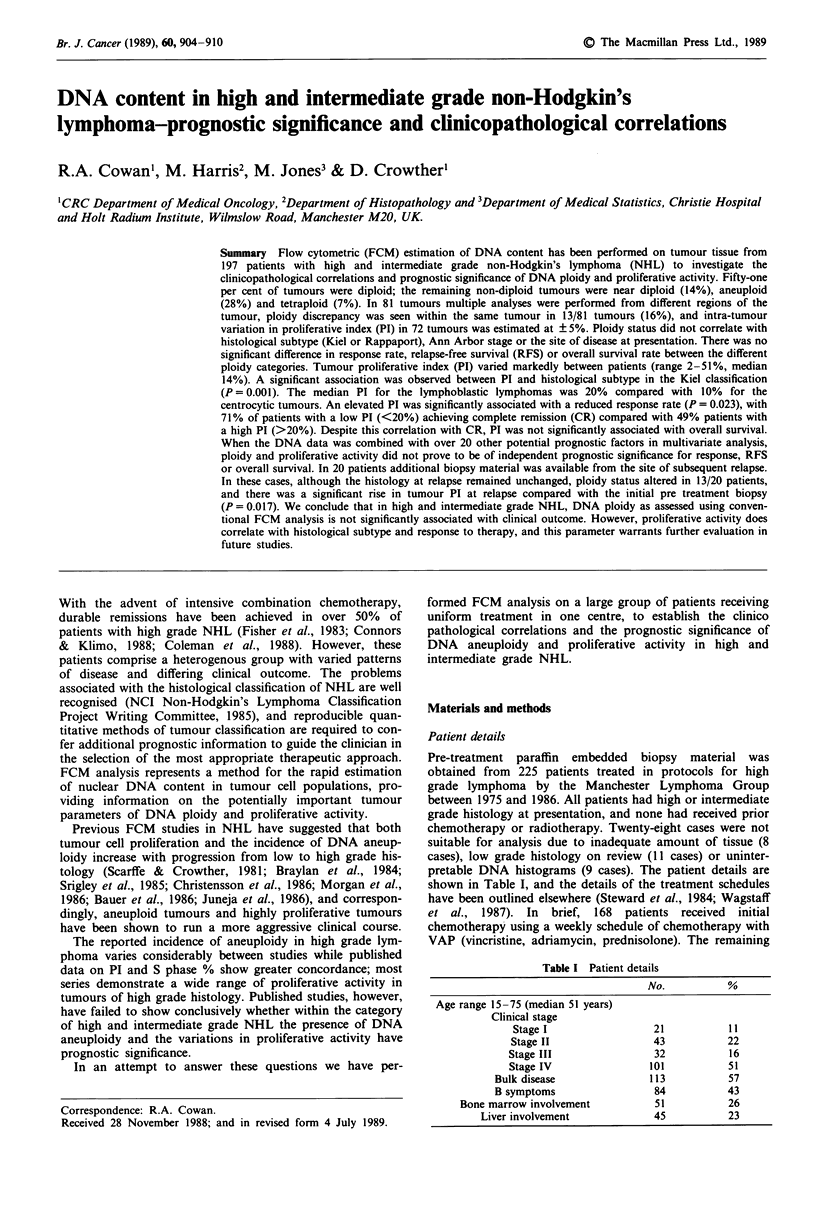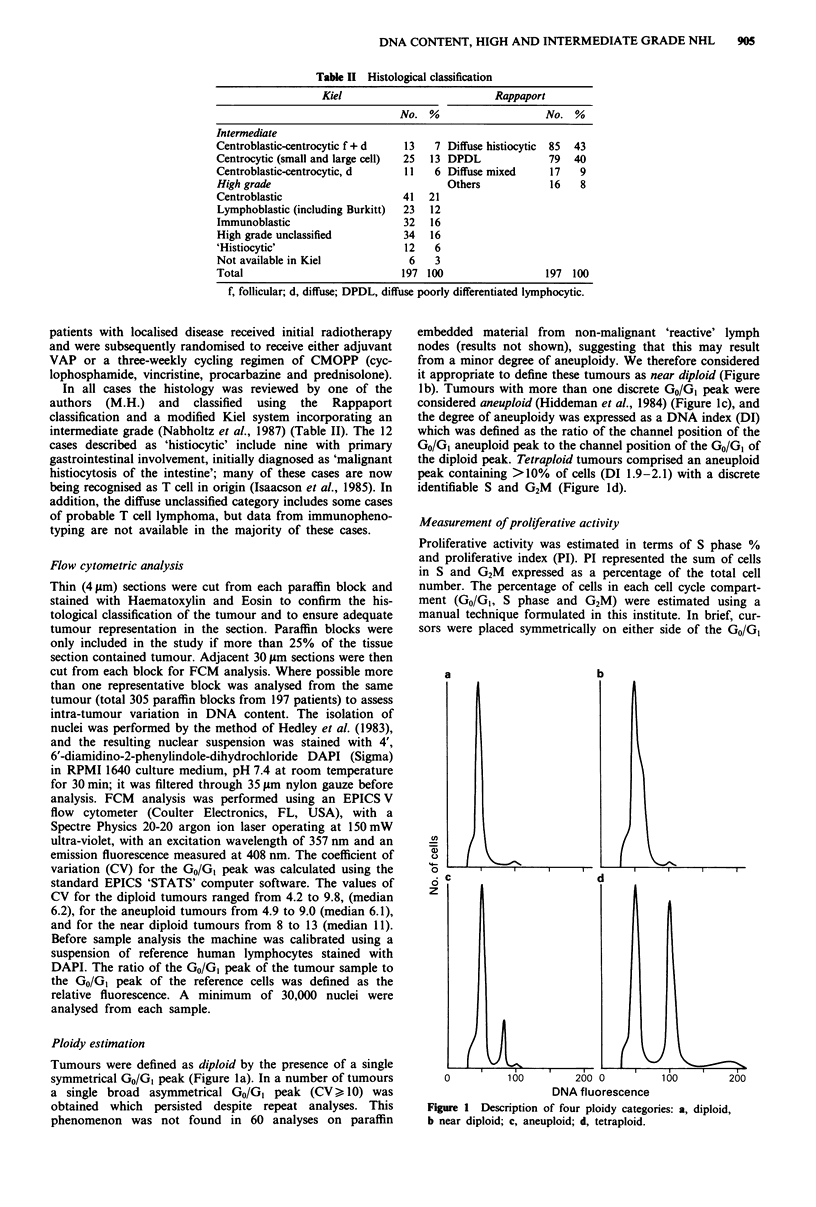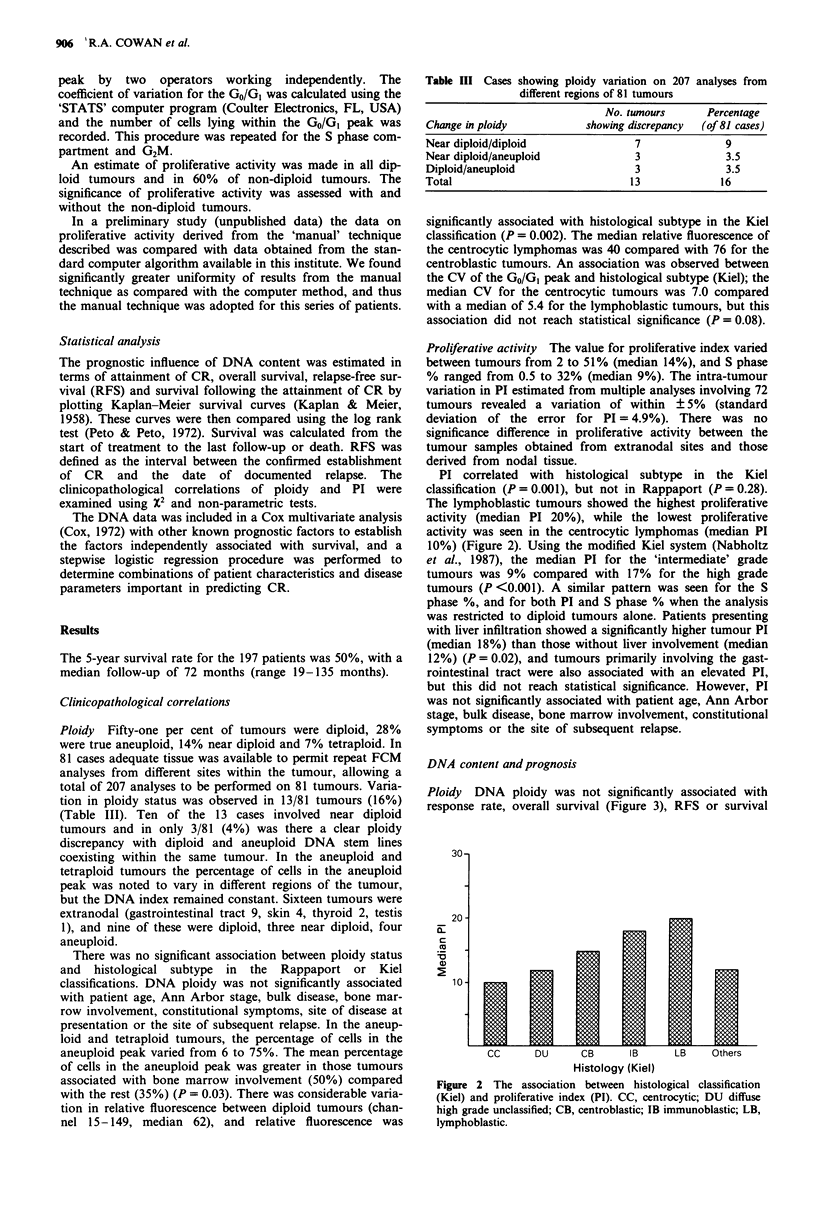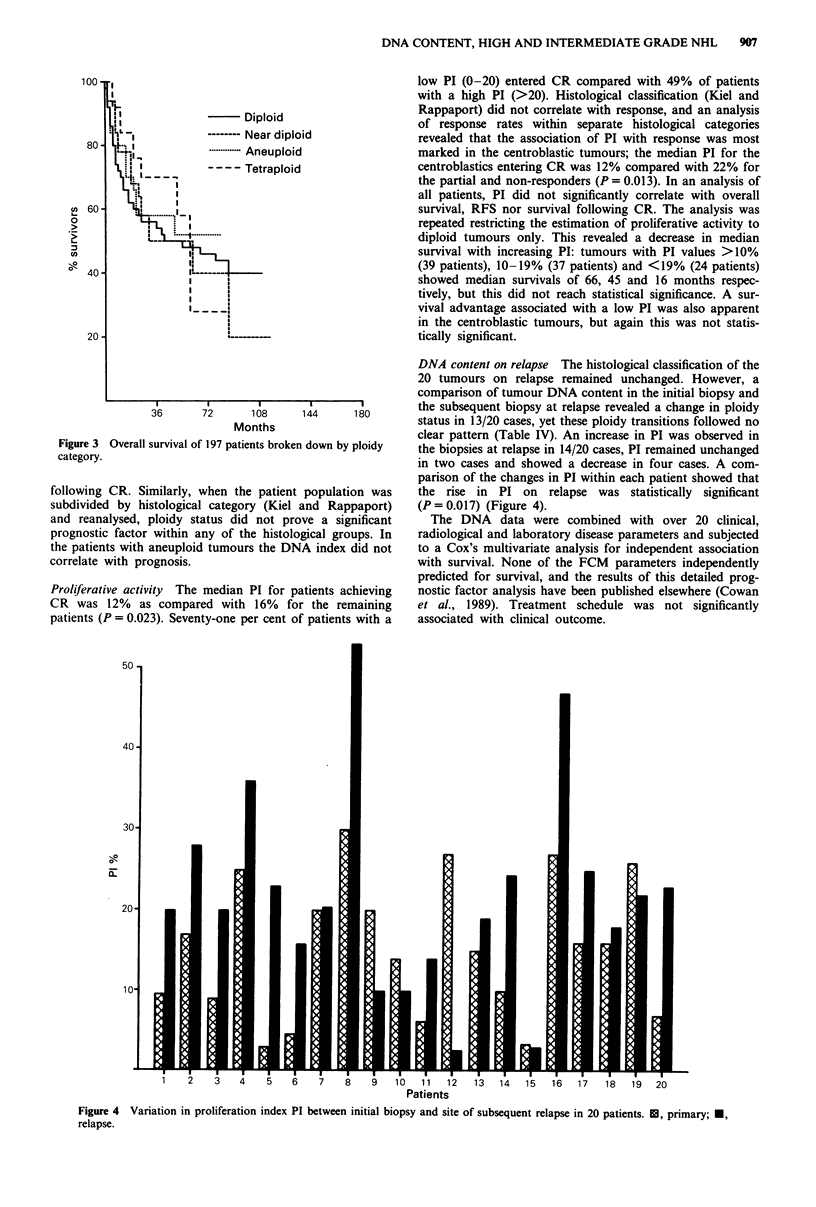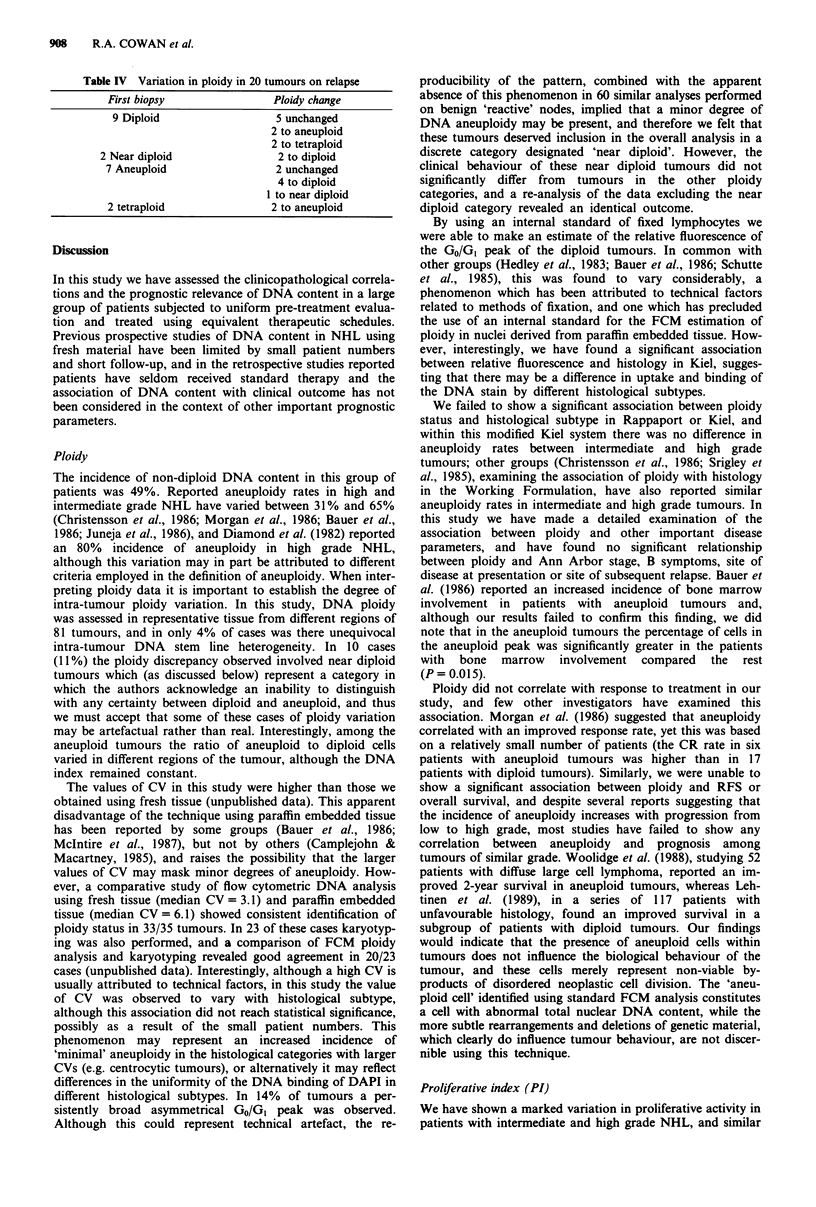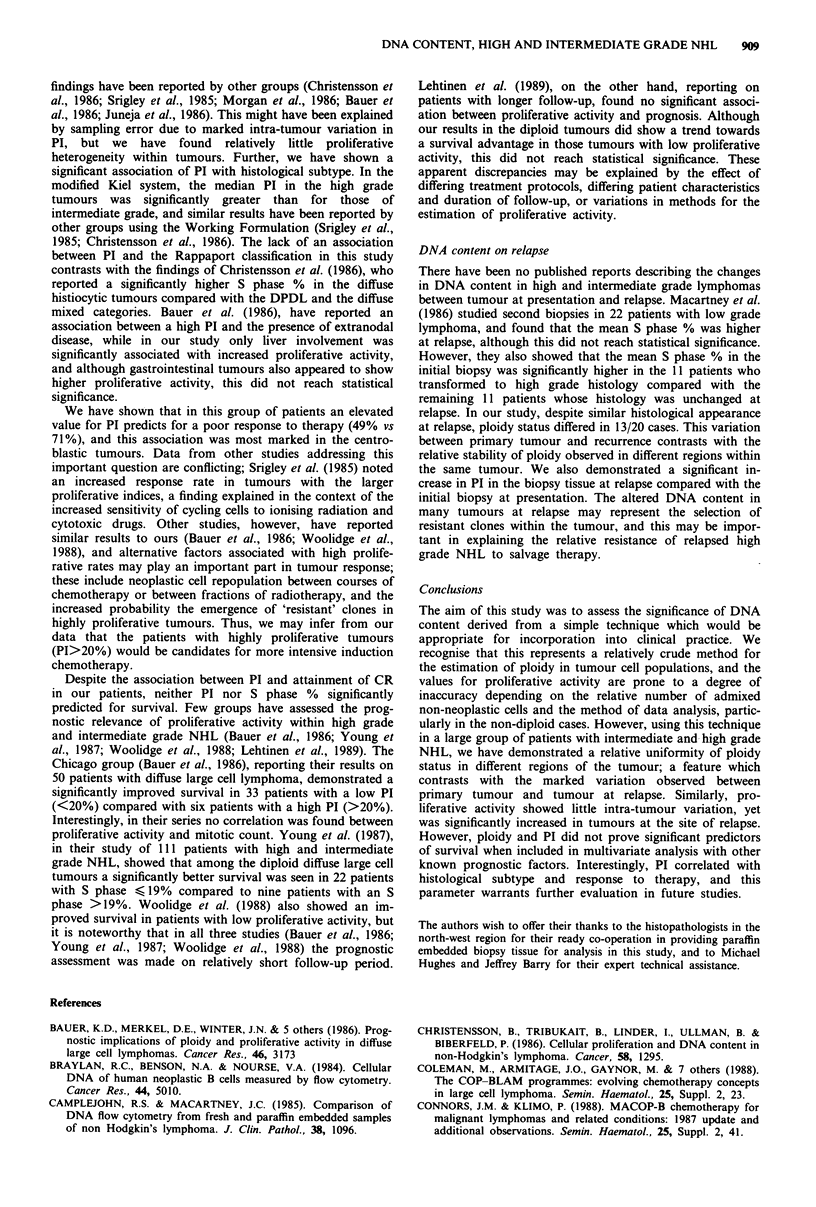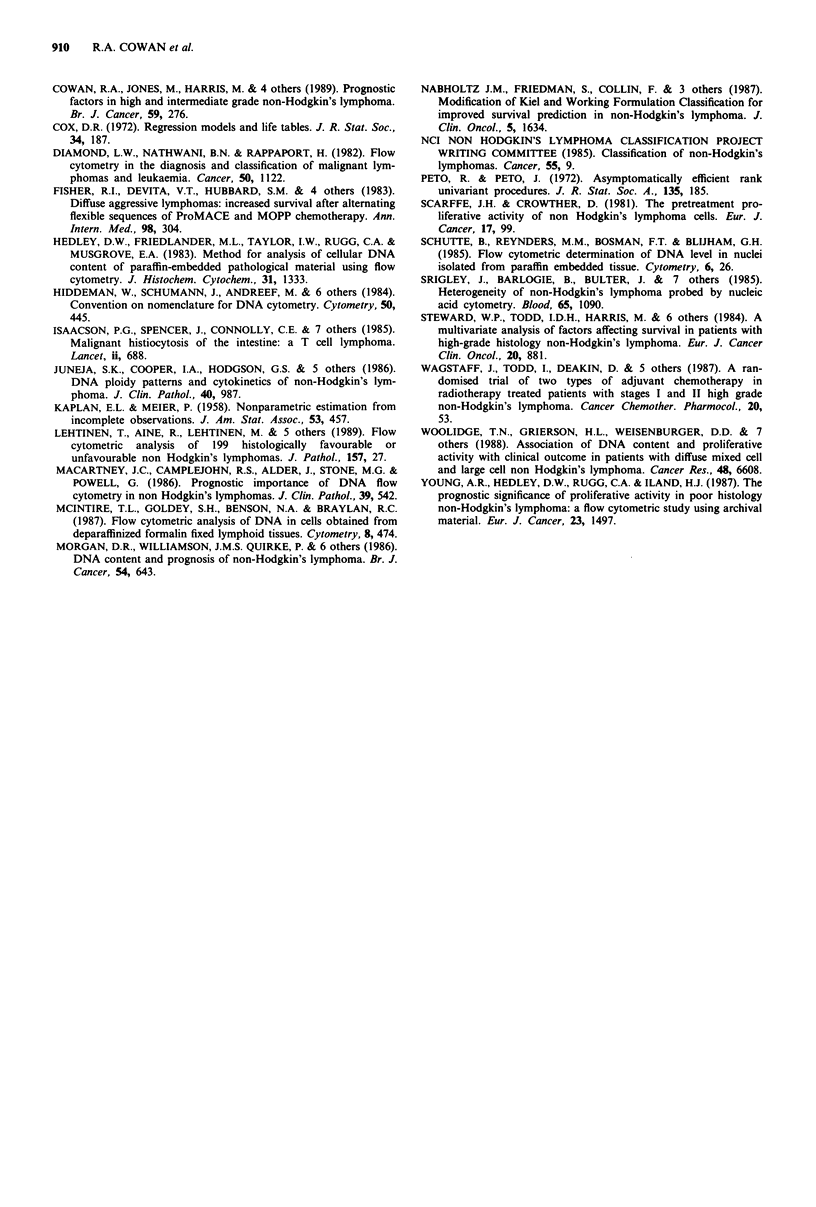# DNA content in high and intermediate grade non-Hodgkin's lymphoma-prognostic significance and clinicopathological correlations.

**DOI:** 10.1038/bjc.1989.388

**Published:** 1989-12

**Authors:** R. A. Cowan, M. Harris, M. Jones, D. Crowther

**Affiliations:** CRC Department of Medical Oncology, Christie Hospital and Holt Radium Institute, Manchester, UK.

## Abstract

Flow cytometric (FCM) estimation of DNA content has been performed on tumour tissue from 197 patients with high and intermediate grade non-Hodgkin's lymphoma (NHL) to investigate the clinicopathological correlations and prognostic significance of DNA ploidy and proliferative activity. Fifty-one per cent of tumours were diploid; the remaining non-diploid tumours were near diploid (14%), aneuploid (28%) and tetraploid (7%). In 81 tumours multiple analyses were performed from different regions of the tumour, ploidy discrepancy was seen within the same tumour in 13/81 tumours (16%), and intra-tumour variation in proliferative index (PI) in 72 tumours was estimated at +/- 5%. Ploidy status did not correlate with histological subtype (Kiel or Rappaport), Ann Arbor stage or the site of disease at presentation. There was no significant difference in response rate, relapse-free survival (RFS) or overall survival rate between the different ploidy categories. Tumour proliferative index (PI) varied markedly between patients (range 2-51%, median 14%). A significant association was observed between PI and histological subtype in the Kiel classification (P = 0.001). The median PI for the lymphoblastic lymphomas was 20% compared with 10% for the centrocytic tumours. An elevated PI was significantly associated with a reduced rate (P = 0.023), with 71% of patients with a low PI (less than 20%) achieving complete remission (CR) compared with 49% patients with a high PI (greater than 20%). Despite this correlation with CR, PI was not significantly associated with overall survival. When the DNA data was combined with over 20 other potential prognostic factors in multivariate analysis, ploidy and proliferative activity did not prove to be of independent prognostic significance for response, RFS or overall survival. In 20 patients additional biopsy material was available from the site of subsequent relapse. In these cases, although the histology at relapse remained unchanged, ploidy status altered in 13/20 patients, and there was a significant rise in tumour PI at relapse compared with the initial pre treatment biopsy (P = 0.017). We conclude that in high and intermediate grade NHL, DNA ploidy as assessed using conventional FCM analysis is not significantly associated with clinical outcome. However, proliferative activity does correlate with histological subtype and response to therapy, and this parameter warrants further evaluation in future studies.


Br. J. Cancer (1989), 60, 904–910 © The Macmillan Press Ltd., 1989

### DNA content in high and intermediate grade non-Hodgkin's lymphoma–prognostic significance and clinicopathological correlations

R.A. Cowan[1], M. Harris[2], M. Jones[3] & D. Crowther[1]

[1]*CRC Department of Medical Oncology,* [2]*Department of Histopathology and* [3]*Department of Medical Statistics, Christie Hospital and Holt Radium Institute, Wilmslow Road, Manchester M20, UK.*

**Summary** Flow cytometric (FCM) estimation of DNA content has been performed on tumour tissue from 197 patients with high and intermediate grade non-Hodgkin's lymphoma (NHL) to investigate the clinicopathological correlations and prognostic significance of DNA ploidy and proliferative activity. Fifty-one per cent of tumours were diploid; the remaining non-diploid tumours were near diploid (14%), aneuploid (28%) and tetraploid (7%). In 81 tumours multiple analyses were performed from different regions of the tumour, ploidy discrepancy was seen within the same tumour in 13/81 tumours (16%), and intra-tumour variation in proliferative index (PI) in 72 tumours was estimated at ±5%. Ploidy status did not correlate with histological subtype (Kiel or Rappaport), Ann Arbor stage or the site of disease at presentation. There was no significant difference in response rate, relapse-free survival (RFS) or overall survival rate between the different ploidy categories. Tumour proliferative index (PI) varied markedly between patients (range 2–51%, median 14%). A significant association was observed between PI and histological subtype in the Kiel classification ($P = 0.001$). The median PI for the lymphoblastic lymphomas was 20% compared with 10% for the centrocytic tumours. An elevated PI was significantly associated with a reduced response rate ($P = 0.023$), with 71% of patients with a low PI (<20%) achieving complete remission (CR) compared with 49% patients with a high PI (>20%). Despite this correlation with CR, PI was not significantly associated with overall survival. When the DNA data was combined with over 20 other potential prognostic factors in multivariate analysis, ploidy and proliferative activity did not prove to be of independent prognostic significance for response, RFS or overall survival. In 20 patients additional biopsy material was available from the site of subsequent relapse. In these cases, although the histology at relapse remained unchanged, ploidy status altered in 13/20 patients, and there was a significant rise in tumour PI at relapse compared with the initial pre treatment biopsy ($P = 0.017$). We conclude that in high and intermediate grade NHL, DNA ploidy as assessed using conventional FCM analysis is not significantly associated with clinical outcome. However, proliferative activity does correlate with histological subtype and response to therapy, and this parameter warrants further evaluation in future studies.

With the advent of intensive combination chemotherapy, durable remissions have been achieved in over 50% of patients with high grade NHL (Fisher *et al.*, 1983; Connors & Klimo, 1988; Coleman *et al.*, 1988). However, these patients comprise a heterogenous group with varied patterns of disease and differing clinical outcome. The problems associated with the histological classification of NHL are well recognised (NCI Non-Hodgkin's Lymphoma Classification Project Writing Committee, 1985), and reproducible quantitative methods of tumour classification are required to confer additional prognostic information to guide the clinician in the selection of the most appropriate therapeutic approach. FCM analysis represents a method for the rapid estimation of nuclear DNA content in tumour cell populations, providing information on the potentially important tumour parameters of DNA ploidy and proliferative activity.

Previous FCM studies in NHL have suggested that both tumour cell proliferation and the incidence of DNA aneuploidy increase with progression from low to high grade histology (Scarffe & Crowther, 1981; Braylan *et al.*, 1984; Srigley *et al.*, 1985; Christensson *et al.*, 1986; Morgan *et al.*, 1986; Bauer *et al.*, 1986; Juneja *et al.*, 1986), and correspondingly, aneuploid tumours and highly proliferative tumours have been shown to run a more aggressive clinical course.

The reported incidence of aneuploidy in high grade lymphoma varies considerably between studies while published data on PI and S phase % show greater concordance; most series demonstrate a wide range of proliferative activity in tumours of high grade histology. Published studies, however, have failed to show conclusively whether within the category of high and intermediate grade NHL the presence of DNA aneuploidy and the variations in proliferative activity have prognostic significance.

In an attempt to answer these questions we have performed FCM analysis on a large group of patients receiving uniform treatment in one centre, to establish the clinico pathological correlations and the prognostic significance of DNA aneuploidy and proliferative activity in high and intermediate grade NHL.

Correspondence: R.A. Cowan.
Received 28 November 1988; and in revised form 4 July 1989.

### Materials and methods

### *Patient details*

Pre-treatment paraffin embedded biopsy material was obtained from 225 patients treated in protocols for high grade lymphoma by the Manchester Lymphoma Group between 1975 and 1986. All patients had high or intermediate grade histology at presentation, and none had received prior chemotherapy or radiotherapy. Twenty-eight cases were not suitable for analysis due to inadequate amount of tissue (8 cases), low grade histology on review (11 cases) or uninterpretable DNA histograms (9 cases). The patient details are shown in Table I, and the details of the treatment schedules have been outlined elsewhere (Steward *et al.*, 1984; Wagstaff *et al.*, 1987). In brief, 168 patients received initial chemotherapy using a weekly schedule of chemotherapy with VAP (vincristine, adriamycin, prednisolone). The remaining

**Table I** Patient details

| | *No.* | *%* |
|---|---|---|
| Age range 15–75 (median 51 years) | | |
| Clinical stage | | |
| Stage I | 21 | 11 |
| Stage II | 43 | 22 |
| Stage III | 32 | 16 |
| Stage IV | 101 | 51 |
| Bulk disease | 113 | 57 |
| B symptoms | 84 | 43 |
| Bone marrow involvement | 51 | 26 |
| Liver involvement | 45 | 23 |

**Table II** Histological classification

| *Kiel* | | | *Rappaport* | | |
|---|---|---|---|---|---|
| | *No.* | *%* | | *No.* | *%* |
| *Intermediate* | | | | | |
| Centroblastic-centrocytic f + d | 13 | 7 | Diffuse histiocytic | 85 | 43 |
| Centrocytic (small and large cell) | 25 | 13 | DPDL | 79 | 40 |
| Centroblastic-centrocytic, d | 11 | 6 | Diffuse mixed | 17 | 9 |
| *High grade* | | | Others | 16 | 8 |
| Centroblastic | 41 | 21 | | | |
| Lymphoblastic (including Burkitt) | 23 | 12 | | | |
| Immunoblastic | 32 | 16 | | | |
| High grade unclassified | 34 | 16 | | | |
| 'Histiocytic' | 12 | 6 | | | |
| Not available in Kiel | 6 | 3 | | | |
| Total | 197 | 100 | | 197 | 100 |

f, follicular; d, diffuse; DPDL, diffuse poorly differentiated lymphocytic.

patients with localised disease received initial radiotherapy and were subsequently randomised to receive either adjuvant VAP or a three-weekly cycling regimen of CMOPP (cyclophosphamide, vincristine, procarbazine and prednisolone).

In all cases the histology was reviewed by one of the authors (M.H.) and classified using the Rappaport classification and a modified Kiel system incorporating an intermediate grade (Nabholtz *et al.*, 1987) (Table II). The 12 cases described as 'histiocytic' include nine with primary gastrointestinal involvement, initially diagnosed as 'malignant histiocytosis of the intestine'; many of these cases are now being recognised as T cell in origin (Isaacson *et al.*, 1985). In addition, the diffuse unclassified category includes some cases of probable T cell lymphoma, but data from immunophenotyping are not available in the majority of these cases.

### *Flow cytometric analysis*

Thin (4 μm) sections were cut from each paraffin block and stained with Haematoxylin and Eosin to confirm the histological classification of the tumour and to ensure adequate tumour representation in the section. Paraffin blocks were only included in the study if more than 25% of the tissue section contained tumour. Adjacent 30 μm sections were then cut from each block for FCM analysis. Where possible more than one representative block was analysed from the same tumour (total 305 paraffin blocks from 197 patients) to assess intra-tumour variation in DNA content. The isolation of nuclei was performed by the method of Hedley *et al.* (1983), and the resulting nuclear suspension was stained with 4′, 6′-diamidino-2-phenylindole-dihydrochloride DAPI (Sigma) in RPMI 1640 culture medium, pH 7.4 at room temperature for 30 min; it was filtered through 35 μm nylon gauze before analysis. FCM analysis was performed using an EPICS V flow cytometer (Coulter Electronics, FL, USA), with a Spectre Physics 20-20 argon ion laser operating at 150 mW ultra-violet, with an excitation wavelength of 357 nm and an emission fluorescence measured at 408 nm. The coefficient of variation (CV) for the $G_0/G_1$ peak was calculated using the standard EPICS 'STATS' computer software. The values of CV for the diploid tumours ranged from 4.2 to 9.8, (median 6.2), for the aneuploid tumours from 4.9 to 9.0 (median 6.1), and for the near diploid tumours from 8 to 13 (median 11). Before sample analysis the machine was calibrated using a suspension of reference human lymphocytes stained with DAPI. The ratio of the $G_0/G_1$ peak of the tumour sample to the $G_0/G_1$ peak of the reference cells was defined as the relative fluorescence. A minimum of 30,000 nuclei were analysed from each sample.

### *Ploidy estimation*

Tumours were defined as *diploid* by the presence of a single symmetrical $G_0/G_1$ peak (Figure 1a). In a number of tumours a single broad asymmetrical $G_0/G_1$ peak ($CV \geqslant 10$) was obtained which persisted despite repeat analyses. This phenomenon was not found in 60 analyses on paraffin embedded material from non-malignant 'reactive' lymph nodes (results not shown), suggesting that this may result from a minor degree of aneuploidy. We therefore considered it appropriate to define these tumours as *near diploid* (Figure 1b). Tumours with more than one discrete $G_0/G_1$ peak were considered *aneuploid* (Hiddeman *et al.*, 1984) (Figure 1c), and the degree of aneuploidy was expressed as a DNA index (DI) which was defined as the ratio of the channel position of the $G_0/G_1$ aneuploid peak to the channel position of the $G_0/G_1$ of the diploid peak. *Tetraploid* tumours comprised an aneuploid peak containing >10% of cells (DI 1.9–2.1) with a discrete identifiable S and $G_2M$ (Figure 1d).

### *Measurement of proliferative activity*

Proliferative activity was estimated in terms of S phase % and proliferative index (PI). PI represented the sum of cells in S and $G_2M$ expressed as a percentage of the total cell number. The percentage of cells in each cell cycle compartment ($G_0/G_1$, S phase and $G_2M$) were estimated using a manual technique formulated in this institute. In brief, cursors were placed symmetrically on either side of the $G_0/G_1$

**Figure 1** Description of four ploidy categories: **a**, diploid, **b** near diploid; **c**, aneuploid; **d**, tetraploid.

peak by two operators working independently. The coefficient of variation for the $G_0/G_1$ was calculated using the 'STATS' computer program (Coulter Electronics, FL, USA) and the number of cells lying within the $G_0/G_1$ peak was recorded. This procedure was repeated for the S phase compartment and $G_2M$.

An estimate of proliferative activity was made in all diploid tumours and in 60% of non-diploid tumours. The significance of proliferative activity was assessed with and without the non-diploid tumours.

In a preliminary study (unpublished data) the data on proliferative activity derived from the 'manual' technique described was compared with data obtained from the standard computer algorithm available in this institute. We found significantly greater uniformity of results from the manual technique as compared with the computer method, and thus the manual technique was adopted for this series of patients.

*Statistical analysis*

The prognostic influence of DNA content was estimated in terms of attainment of CR, overall survival, relapse-free survival (RFS) and survival following the attainment of CR by plotting Kaplan–Meier survival curves (Kaplan & Meier, 1958). These curves were then compared using the log rank test (Peto & Peto, 1972). Survival was calculated from the start of treatment to the last follow-up or death. RFS was defined as the interval between the confirmed establishment of CR and the date of documented relapse. The clinicopathological correlations of ploidy and PI were examined using $\chi^2$ and non-parametric tests.

The DNA data was included in a Cox multivariate analysis (Cox, 1972) with other known prognostic factors to establish the factors independently associated with survival, and a stepwise logistic regression procedure was performed to determine combinations of patient characteristics and disease parameters important in predicting CR.

### Results

The 5-year survival rate for the 197 patients was 50%, with a median follow-up of 72 months (range 19–135 months).

*Clinicopathological correlations*

*Ploidy* Fifty-one per cent of tumours were diploid, 28% were true aneuploid, 14% near diploid and 7% tetraploid. In 81 cases adequate tissue was available to permit repeat FCM analyses from different sites within the tumour, allowing a total of 207 analyses to be performed on 81 tumours. Variation in ploidy status was observed in 13/81 tumours (16%) (Table III). Ten of the 13 cases involved near diploid tumours and in only 3/81 (4%) was there a clear ploidy discrepancy with diploid and aneuploid DNA stem lines coexisting within the same tumour. In the aneuploid and tetraploid tumours the percentage of cells in the aneuploid peak was noted to vary in different regions of the tumour, but the DNA index remained constant. Sixteen tumours were extranodal (gastrointestinal tract 9, skin 4, thyroid 2, testis 1), and nine of these were diploid, three near diploid, four aneuploid.

There was no significant association between ploidy status and histological subtype in the Rappaport or Kiel classifications. DNA ploidy was not significantly associated with patient age, Ann Arbor stage, bulk disease, bone marrow involvement, constitutional symptoms, site of disease at presentation or the site of subsequent relapse. In the aneuploid and tetraploid tumours, the percentage of cells in the aneuploid peak varied from 6 to 75%. The mean percentage of cells in the aneuploid peak was greater in those tumours associated with bone marrow involvement (50%) compared with the rest (35%) ($P = 0.03$). There was considerable variation in relative fluorescence between diploid tumours (channel 15–149, median 62), and relative fluorescence was

**Table III** Cases showing ploidy variation on 207 analyses from different regions of 81 tumours

| *Change in ploidy* | *No. tumours showing discrepancy* | *Percentage (of 81 cases)* |
|---|---|---|
| Near diploid/diploid | 7 | 9 |
| Near diploid/aneuploid | 3 | 3.5 |
| Diploid/aneuploid | 3 | 3.5 |
| Total | 13 | 16 |

significantly associated with histological subtype in the Kiel classification ($P = 0.002$). The median relative fluorescence of the centrocytic lymphomas was 40 compared with 76 for the centroblastic tumours. An association was observed between the CV of the $G_0/G_1$ peak and histological subtype (Kiel); the median CV for the centrocytic tumours was 7.0 compared with a median of 5.4 for the lymphoblastic tumours, but this association did not reach statistical significance ($P = 0.08$).

*Proliferative activity* The value for proliferative index varied between tumours from 2 to 51% (median 14%), and S phase % ranged from 0.5 to 32% (median 9%). The intra-tumour variation in PI estimated from multiple analyses involving 72 tumours revealed a variation of within ±5% (standard deviation of the error for PI = 4.9%). There was no significance difference in proliferative activity between the tumour samples obtained from extranodal sites and those derived from nodal tissue.

PI correlated with histological subtype in the Kiel classification ($P = 0.001$), but not in Rappaport ($P = 0.28$). The lymphoblastic tumours showed the highest proliferative activity (median PI 20%), while the lowest proliferative activity was seen in the centrocytic lymphomas (median PI 10%) (Figure 2). Using the modified Kiel system (Nabholtz *et al.*, 1987), the median PI for the 'intermediate' grade tumours was 9% compared with 17% for the high grade tumours ($P < 0.001$). A similar pattern was seen for the S phase %, and for both PI and S phase % when the analysis was restricted to diploid tumours alone. Patients presenting with liver infiltration showed a significantly higher tumour PI (median 18%) than those without liver involvement (median 12%) ($P = 0.02$), and tumours primarily involving the gastrointestinal tract were also associated with an elevated PI, but this did not reach statistical significance. However, PI was not significantly associated with patient age, Ann Arbor stage, bulk disease, bone marrow involvement, constitutional symptoms or the site of subsequent relapse.

*DNA content and prognosis*

*Ploidy* DNA ploidy was not significantly associated with response rate, overall survival (Figure 3), RFS or survival

**Figure 2** The association between histological classification (Kiel) and proliferative index (PI). CC, centrocytic; DU diffuse high grade unclassified; CB, centroblastic; IB immunoblastic; LB, lymphoblastic.

**Figure 3** Overall survival of 197 patients broken down by ploidy category.

following CR. Similarly, when the patient population was subdivided by histological category (Kiel and Rappaport) and reanalysed, ploidy status did not prove a significant prognostic factor within any of the histological groups. In the patients with aneuploid tumours the DNA index did not correlate with prognosis.

*Proliferative activity* The median PI for patients achieving CR was 12% as compared with 16% for the remaining patients ($P = 0.023$). Seventy-one per cent of patients with a low PI (0–20) entered CR compared with 49% of patients with a high PI (>20). Histological classification (Kiel and Rappaport) did not correlate with response, and an analysis of response rates within separate histological categories revealed that the association of PI with response was most marked in the centroblastic tumours; the median PI for the centroblastics entering CR was 12% compared with 22% for the partial and non-responders ($P = 0.013$). In an analysis of all patients, PI did not significantly correlate with overall survival, RFS nor survival following CR. The analysis was repeated restricting the estimation of proliferative activity to diploid tumours only. This revealed a decrease in median survival with increasing PI: tumours with PI values >10% (39 patients), 10–19% (37 patients) and <19% (24 patients) showed median survivals of 66, 45 and 16 months respectively, but this did not reach statistical significance. A survival advantage associated with a low PI was also apparent in the centroblastic tumours, but again this was not statistically significant.

*DNA content on relapse* The histological classification of the 20 tumours on relapse remained unchanged. However, a comparison of tumour DNA content in the initial biopsy and the subsequent biopsy at relapse revealed a change in ploidy status in 13/20 cases, yet these ploidy transitions followed no clear pattern (Table IV). An increase in PI was observed in the biopsies at relapse in 14/20 cases, PI remained unchanged in two cases and showed a decrease in four cases. A comparison of the changes in PI within each patient showed that the rise in PI on relapse was statistically significant ($P = 0.017$) (Figure 4).

The DNA data were combined with over 20 clinical, radiological and laboratory disease parameters and subjected to a Cox's multivariate analysis for independent association with survival. None of the FCM parameters independently predicted for survival, and the results of this detailed prognostic factor analysis have been published elsewhere (Cowan *et al.*, 1989). Treatment schedule was not significantly associated with clinical outcome.

**Figure 4** Variation in proliferation index PI between initial biopsy and site of subsequent relapse in 20 patients. ▨, primary; ■, relapse.

Table IV Variation in ploidy in 20 tumours on relapse

| First biopsy | Ploidy change |
|---|---|
| 9 Diploid | 5 unchanged |
| | 2 to aneuploid |
| | 2 to tetraploid |
| 2 Near diploid | 2 to diploid |
| 7 Aneuploid | 2 unchanged |
| | 4 to diploid |
| | 1 to near diploid |
| 2 tetraploid | 2 to aneuploid |

### Discussion

In this study we have assessed the clinicopathological correlations and the prognostic relevance of DNA content in a large group of patients subjected to uniform pre-treatment evaluation and treated using equivalent therapeutic schedules. Previous prospective studies of DNA content in NHL using fresh material have been limited by small patient numbers and short follow-up, and in the retrospective studies reported patients have seldom received standard therapy and the association of DNA content with clinical outcome has not been considered in the context of other important prognostic parameters.

### *Ploidy*

The incidence of non-diploid DNA content in this group of patients was 49%. Reported aneuploidy rates in high and intermediate grade NHL have varied between 31% and 65% (Christensson *et al.*, 1986; Morgan *et al.*, 1986; Bauer *et al.*, 1986; Juneja *et al.*, 1986), and Diamond *et al.* (1982) reported an 80% incidence of aneuploidy in high grade NHL, although this variation may in part be attributed to different criteria employed in the definition of aneuploidy. When interpreting ploidy data it is important to establish the degree of intra-tumour ploidy variation. In this study, DNA ploidy was assessed in representative tissue from different regions of 81 tumours, and in only 4% of cases was there unequivocal intra-tumour DNA stem line heterogeneity. In 10 cases (11%) the ploidy discrepancy observed involved near diploid tumours which (as discussed below) represent a category in which the authors acknowledge an inability to distinguish with any certainty between diploid and aneuploid, and thus we must accept that some of these cases of ploidy variation may be artefactual rather than real. Interestingly, among the aneuploid tumours the ratio of aneuploid to diploid cells varied in different regions of the tumour, although the DNA index remained constant.

The values of CV in this study were higher than those we obtained using fresh tissue (unpublished data). This apparent disadvantage of the technique using paraffin embedded tissue has been reported by some groups (Bauer *et al.*, 1986; McIntire *et al.*, 1987), but not by others (Camplejohn & Macartney, 1985), and raises the possibility that the larger values of CV may mask minor degrees of aneuploidy. However, a comparative study of flow cytometric DNA analysis using fresh tissue (median CV = 3.1) and paraffin embedded tissue (median CV = 6.1) showed consistent identification of ploidy status in 33/35 tumours. In 23 of these cases karyotyping was also performed, and a comparison of FCM ploidy analysis and karyotyping revealed good agreement in 20/23 cases (unpublished data). Interestingly, although a high CV is usually attributed to technical factors, in this study the value of CV was observed to vary with histological subtype, although this association did not reach statistical significance, possibly as a result of the small patient numbers. This phenomenon may represent an increased incidence of 'minimal' aneuploidy in the histological categories with larger CVs (e.g. centrocytic tumours), or alternatively it may reflect differences in the uniformity of the DNA binding of DAPI in different histological subtypes. In 14% of tumours a persistently broad asymmetrical $G_0/G_1$ peak was observed. Although this could represent technical artefact, the reproducibility of the pattern, combined with the apparent absence of this phenomenon in 60 similar analyses performed on benign 'reactive' nodes, implied that a minor degree of DNA aneuploidy may be present, and therefore we felt that these tumours deserved inclusion in the overall analysis in a discrete category designated 'near diploid'. However, the clinical behaviour of these near diploid tumours did not significantly differ from tumours in the other ploidy categories, and a re-analysis of the data excluding the near diploid category revealed an identical outcome.

By using an internal standard of fixed lymphocytes we were able to make an estimate of the relative fluorescence of the $G_0/G_1$ peak of the diploid tumours. In common with other groups (Hedley *et al.*, 1983; Bauer *et al.*, 1986; Schutte *et al.*, 1985), this was found to vary considerably, a phenomenon which has been attributed to technical factors related to methods of fixation, and one which has precluded the use of an internal standard for the FCM estimation of ploidy in nuclei derived from paraffin embedded tissue. However, interestingly, we have found a significant association between relative fluorescence and histology in Kiel, suggesting that there may be a difference in uptake and binding of the DNA stain by different histological subtypes.

We failed to show a significant association between ploidy status and histological subtype in Rappaport or Kiel, and within this modified Kiel system there was no difference in aneuploidy rates between intermediate and high grade tumours; other groups (Christensson *et al.*, 1986; Srigley *et al.*, 1985), examining the association of ploidy with histology in the Working Formulation, have also reported similar aneuploidy rates in intermediate and high grade tumours. In this study we have made a detailed examination of the association between ploidy and other important disease parameters, and have found no significant relationship between ploidy and Ann Arbor stage, B symptoms, site of disease at presentation or site of subsequent relapse. Bauer *et al.* (1986) reported an increased incidence of bone marrow involvement in patients with aneuploid tumours and, although our results failed to confirm this finding, we did note that in the aneuploid tumours the percentage of cells in the aneuploid peak was significantly greater in the patients with bone marrow involvement compared the rest ($P = 0.015$).

Ploidy did not correlate with response to treatment in our study, and few other investigators have examined this association. Morgan *et al.* (1986) suggested that aneuploidy correlated with an improved response rate, yet this was based on a relatively small number of patients (the CR rate in six patients with aneuploid tumours was higher than in 17 patients with diploid tumours). Similarly, we were unable to show a significant association between ploidy and RFS or overall survival, and despite several reports suggesting that the incidence of aneuploidy increases with progression from low to high grade, most studies have failed to show any correlation between aneuploidy and prognosis among tumours of similar grade. Woolidge *et al.* (1988), studying 52 patients with diffuse large cell lymphoma, reported an improved 2-year survival in aneuploid tumours, whereas Lehtinen *et al.* (1989), in a series of 117 patients with unfavourable histology, found an improved survival in a subgroup of patients with diploid tumours. Our findings would indicate that the presence of aneuploid cells within tumours does not influence the biological behaviour of the tumour, and these cells merely represent non-viable by-products of disordered neoplastic cell division. The 'aneuploid cell' identified using standard FCM analysis constitutes a cell with abnormal total nuclear DNA content, while the more subtle rearrangements and deletions of genetic material, which clearly do influence tumour behaviour, are not discernible using this technique.

### *Proliferative index (PI)*

We have shown a marked variation in proliferative activity in patients with intermediate and high grade NHL, and similar findings have been reported by other groups (Christensson *et al.*, 1986; Srigley *et al.*, 1985; Morgan *et al.*, 1986; Bauer *et al.*, 1986; Juneja *et al.*, 1986). This might have been explained by sampling error due to marked intra-tumour variation in PI, but we have found relatively little proliferative heterogeneity within tumours. Further, we have shown a significant association of PI with histological subtype. In the modified Kiel system, the median PI in the high grade tumours was significantly greater than for those of intermediate grade, and similar results have been reported by other groups using the Working Formulation (Srigley *et al.*, 1985; Christensson *et al.*, 1986). The lack of an association between PI and the Rappaport classification in this study contrasts with the findings of Christensson *et al.* (1986), who reported a significantly higher S phase % in the diffuse histiocytic tumours compared with the DPDL and the diffuse mixed categories. Bauer *et al.* (1986), have reported an association between a high PI and the presence of extranodal disease, while in our study only liver involvement was significantly associated with increased proliferative activity, and although gastrointestinal tumours also appeared to show higher proliferative activity, this did not reach statistical significance.

We have shown that in this group of patients an elevated value for PI predicts for a poor response to therapy (49% *vs* 71%), and this association was most marked in the centroblastic tumours. Data from other studies addressing this important question are conflicting; Srigley *et al.* (1985) noted an increased response rate in tumours with the larger proliferative indices, a finding explained in the context of the increased sensitivity of cycling cells to ionising radiation and cytotoxic drugs. Other studies, however, have reported similar results to ours (Bauer *et al.*, 1986; Woolidge *et al.*, 1988), and alternative factors associated with high proliferative rates may play an important part in tumour response; these include neoplastic cell repopulation between courses of chemotherapy or between fractions of radiotherapy, and the increased probability the emergence of 'resistant' clones in highly proliferative tumours. Thus, we may infer from our data that the patients with highly proliferative tumours (PI>20%) would be candidates for more intensive induction chemotherapy.

Despite the association between PI and attainment of CR in our patients, neither PI nor S phase % significantly predicted for survival. Few groups have assessed the prognostic relevance of proliferative activity within high grade and intermediate grade NHL (Bauer *et al.*, 1986; Young *et al.*, 1987; Woolidge *et al.*, 1988; Lehtinen *et al.*, 1989). The Chicago group (Bauer *et al.*, 1986), reporting their results on 50 patients with diffuse large cell lymphoma, demonstrated a significantly improved survival in 33 patients with a low PI (<20%) compared with six patients with a high PI (>20%). Interestingly, in their series no correlation was found between proliferative activity and mitotic count. Young *et al.* (1987), in their study of 111 patients with high and intermediate grade NHL, showed that among the diploid diffuse large cell tumours a significantly better survival was seen in 22 patients with S phase $\leqslant$19% compared to nine patients with an S phase >19%. Woolidge *et al.* (1988) also showed an improved survival in patients with low proliferative activity, but it is noteworthy that in all three studies (Bauer *et al.*, 1986; Young *et al.*, 1987; Woolidge *et al.*, 1988) the prognostic assessment was made on relatively short follow-up period. Lehtinen *et al.* (1989), on the other hand, reporting on patients with longer follow-up, found no significant association between proliferative activity and prognosis. Although our results in the diploid tumours did show a trend towards a survival advantage in those tumours with low proliferative activity, this did not reach statistical significance. These apparent discrepancies may be explained by the effect of differing treatment protocols, differing patient characteristics and duration of follow-up, or variations in methods for the estimation of proliferative activity.

### *DNA content on relapse*

There have been no published reports describing the changes in DNA content in high and intermediate grade lymphomas between tumour at presentation and relapse. Macartney *et al.* (1986) studied second biopsies in 22 patients with low grade lymphoma, and found that the mean S phase % was higher at relapse, although this did not reach statistical significance. However, they also showed that the mean S phase % in the initial biopsy was significantly higher in the 11 patients who transformed to high grade histology compared with the remaining 11 patients whose histology was unchanged at relapse. In our study, despite similar histological appearance at relapse, ploidy status differed in 13/20 cases. This variation between primary tumour and recurrence contrasts with the relative stability of ploidy observed in different regions within the same tumour. We also demonstrated a significant increase in PI in the biopsy tissue at relapse compared with the initial biopsy at presentation. The altered DNA content in many tumours at relapse may represent the selection of resistant clones within the tumour, and this may be important in explaining the relative resistance of relapsed high grade NHL to salvage therapy.

### *Conclusions*

The aim of this study was to assess the significance of DNA content derived from a simple technique which would be appropriate for incorporation into clinical practice. We recognise that this represents a relatively crude method for the estimation of ploidy in tumour cell populations, and the values for proliferative activity are prone to a degree of inaccuracy depending on the relative number of admixed non-neoplastic cells and the method of data analysis, particularly in the non-diploid cases. However, using this technique in a large group of patients with intermediate and high grade NHL, we have demonstrated a relative uniformity of ploidy status in different regions of the tumour; a feature which contrasts with the marked variation observed between primary tumour and tumour at relapse. Similarly, proliferative activity showed little intra-tumour variation, yet was significantly increased in tumours at the site of relapse. However, ploidy and PI did not prove significant predictors of survival when included in multivariate analysis with other known prognostic factors. Interestingly, PI correlated with histological subtype and response to therapy, and this parameter warrants further evaluation in future studies.

The authors wish to offer their thanks to the histopathologists in the north-west region for their ready co-operation in providing paraffin embedded biopsy tissue for analysis in this study, and to Michael Hughes and Jeffrey Barry for their expert technical assistance.

### References

BAUER, K.D., MERKEL, D.E., WINTER, J.N. & 5 others (1986). Prognostic implications of ploidy and proliferative activity in diffuse large cell lymphomas. *Cancer Res.*, **46**, 3173

BRAYLAN, R.C., BENSON, N.A. & NOURSE, V.A. (1984). Cellular DNA of human neoplastic B cells measured by flow cytometry. *Cancer Res.*, **44**, 5010.

CAMPLEJOHN, R.S. & MACARTNEY, J.C. (1985). Comparison of DNA flow cytometry from fresh and paraffin embedded samples of non Hodgkin's lymphoma. *J. Clin. Pathol.*, **38**, 1096.

CHRISTENSSON, B., TRIBUKAIT, B., LINDER, I., ULLMAN, B. & BIBERFELD, P. (1986). Cellular proliferation and DNA content in non-Hodgkin's lymphoma. *Cancer*, **58**, 1295.

COLEMAN, M., ARMITAGE, J.O., GAYNOR, M. & 7 others (1988). The COP–BLAM programmes: evolving chemotherapy concepts in large cell lymphoma. *Semin. Haematol.*, **25**, Suppl. 2, 23.

CONNORS, J.M. & KLIMO, P. (1988). MACOP-B chemotherapy for malignant lymphomas and related conditions: 1987 update and additional observations. *Semin. Haematol.*, **25**, Suppl. 2, 41.

COWAN, R.A., JONES, M., HARRIS, M. & 4 others (1989). Prognostic factors in high and intermediate grade non-Hodgkin's lymphoma. *Br. J. Cancer*, **59**, 276.

COX, D.R. (1972). Regression models and life tables. *J. R. Stat. Soc.*, **34**, 187.

DIAMOND, L.W., NATHWANI, B.N. & RAPPAPORT, H. (1982). Flow cytometry in the diagnosis and classification of malignant lymphomas and leukaemia. *Cancer*, **50**, 1122.

FISHER, R.I., DEVITA, V.T., HUBBARD, S.M. & 4 others (1983). Diffuse aggressive lymphomas: increased survival after alternating flexible sequences of ProMACE and MOPP chemotherapy. *Ann. Intern. Med.*, **98**, 304.

HEDLEY, D.W., FRIEDLANDER, M.L., TAYLOR, I.W., RUGG, C.A. & MUSGROVE, E.A. (1983). Method for analysis of cellular DNA content of paraffin-embedded pathological material using flow cytometry. *J. Histochem. Cytochem.*, **31**, 1333.

HIDDEMAN, W., SCHUMANN, J., ANDREEF, M. & 6 others (1984). Convention on nomenclature for DNA cytometry. *Cytometry*, **50**, 445.

ISAACSON, P.G., SPENCER, J., CONNOLLY, C.E. & 7 others (1985). Malignant histiocytosis of the intestine: a T cell lymphoma. *Lancet*, **ii**, 688.

JUNEJA, S.K., COOPER, I.A., HODGSON, G.S. & 5 others (1986). DNA ploidy patterns and cytokinetics of non-Hodgkin's lymphoma. *J. Clin. Pathol.*, **40**, 987.

KAPLAN, E.L. & MEIER, P. (1958). Nonparametric estimation from incomplete observations. *J. Am. Stat. Assoc.*, **53**, 457.

LEHTINEN, T., AINE, R., LEHTINEN, M. & 5 others (1989). Flow cytometric analysis of 199 histologically favourable or unfavourable non Hodgkin's lymphomas. *J. Pathol.*, **157**, 27.

MACARTNEY, J.C., CAMPLEJOHN, R.S., ALDER, J., STONE, M.G. & POWELL, G. (1986). Prognostic importance of DNA flow cytometry in non Hodgkin's lymphomas. *J. Clin. Pathol.*, **39**, 542.

MCINTIRE, T.L., GOLDEY, S.H., BENSON, N.A. & BRAYLAN, R.C. (1987). Flow cytometric analysis of DNA in cells obtained from deparaffinized formalin fixed lymphoid tissues. *Cytometry*, **8**, 474.

MORGAN, D.R., WILLIAMSON, J.M.S. QUIRKE, P. & 6 others (1986). DNA content and prognosis of non-Hodgkin's lymphoma. *Br. J. Cancer*, **54**, 643.

NABHOLTZ J.M., FRIEDMAN, S., COLLIN, F. & 3 others (1987). Modification of Kiel and Working Formulation Classification for improved survival prediction in non-Hodgkin's lymphoma. *J. Clin. Oncol.*, **5**, 1634.

NCI NON HODGKIN'S LYMPHOMA CLASSIFICATION PROJECT WRITING COMMITTEE (1985). Classification of non-Hodgkin's lymphomas. *Cancer*, **55**, 9.

PETO, R. & PETO, J. (1972). Asymptomatically efficient rank univariant procedures. *J. R. Stat. Soc. A.*, **135**, 185.

SCARFFE, J.H. & CROWTHER, D. (1981). The pretreatment proliferative activity of non Hodgkin's lymphoma cells. *Eur. J. Cancer*, **17**, 99.

SCHUTTE, B., REYNDERS, M.M., BOSMAN, F.T. & BLIJHAM, G.H. (1985). Flow cytometric determination of DNA level in nuclei isolated from paraffin embedded tissue. *Cytometry*, **6**, 26.

SRIGLEY, J., BARLOGIE, B., BULTER, J. & 7 others (1985). Heterogeneity of non-Hodgkin's lymphoma probed by nucleic acid cytometry. *Blood*, **65**, 1090.

STEWARD, W.P., TODD, I.D.H., HARRIS, M. & 6 others (1984). A multivariate analysis of factors affecting survival in patients with high-grade histology non-Hodgkin's lymphoma. *Eur. J. Cancer Clin. Oncol.*, **20**, 881.

WAGSTAFF, J., TODD, I., DEAKIN, D. & 5 others (1987). A randomised trial of two types of adjuvant chemotherapy in radiotherapy treated patients with stages I and II high grade non-Hodgkin's lymphoma. *Cancer Chemother. Pharmocol.*, **20**, 53.

WOOLIDGE, T.N., GRIERSON, H.L., WEISENBURGER, D.D. & 7 others (1988). Association of DNA content and proliferative activity with clinical outcome in patients with diffuse mixed cell and large cell non Hodgkin's lymphoma. *Cancer Res.*, **48**, 6608.

YOUNG, A.R., HEDLEY, D.W., RUGG, C.A. & ILAND, H.J. (1987). The prognostic significance of proliferative activity in poor histology non-Hodgkin's lymphoma: a flow cytometric study using archival material. *Eur. J. Cancer*, **23**, 1497.